# Medical Book-Keeping

**Published:** 1891-02-07

**Authors:** 


### MEDICAL BOOK-KEEPING.

The young practitioner, having selected a locality in which to practise, very quickly finds that he must decide upon a method of recording the work he does, and the medicines he dispenses. He chooses a method from amongst the many in use, and regularly records his professional work in a daybook for the purpose of posting every week or month, and delivering quarterly accounts to his patients. with a greater or less prospect of having a "short reckoning" for them. This is his duty to his patients, and a very important duty; one which, it is a well-known fact, is very sadly neglected. This may be accounted for by the many heavy calls upon a doctor's time. When the visiting is done there is little inclination to sit down to the ledger, and the posting, therefore, by many medical men is left for weeks—frequently until the end of the half-year; whilst patients, anxious to pay, clamour for their accounts until they get tired of asking for them. The various methods have been very fully discussed quite recently in the *British Medical Journal*, and, in discharging his duty to his patients, it will be the business of each practitioner to make himself acquainted with them, and select that which, taken all round, seems best to suit the requirements of general practice.

The only plan by which a medical man can successfully appeal against an assessment which he knows to be too high, is by keeping a cash-book. In this way only is he enabled to do his duty to himself. From its pages he, in a very short space of time, prepares a debit and credit account, with the proper expenses all deducted, to show plainly and unquestionably at what figure his assessment should really stand.

This being the case, it is with pleasure we notice that Dr. Illingworth, of Accrington, has designed a cash-book especially for medical practitioners, in which indications are fully given for the accurate rendering of statements required by all medical men in practice, and for the preparation of income-tax returns.

So far as we are aware, this is the first cash-book which has been issued for the special use of medical men, and we are sure it will be welcomed by many who have already suffered from the worry and annoyance of preparing income-tax returns to no purpose except disappointment. The cash-book is manufactured and sold by Messrs. Broadley, publishers, Clayton, near Accrington.

